# Adjuvant Chemotherapy in pT2N0M0 Gastric Cancer: Findings From a Retrospective Study

**DOI:** 10.3389/fphar.2022.845261

**Published:** 2022-02-17

**Authors:** Yu Mei, Xijia Feng, Tienan Feng, Min Yan, Zhenggang Zhu, Tian Li, Zhenglun Zhu

**Affiliations:** ^1^ Department of General Surgery, Gastrointestinal Surgery, Shanghai Key Laboratory of Gastric Neoplasms, Shanghai Institute of Digestive Surgery, Ruijin Hospital, Shanghai Jiao Tong University School of Medicine, Shanghai, China; ^2^ Department of Thoracic Surgery, Ruijin Hospital, Shanghai Jiao Tong University School of Medicine, Shanghai, China; ^3^ Clinical Research Institute, Shanghai Jiao Tong University School of Medicine, Shanghai, China; ^4^ School of Basic Medicine, Fourth Military Medical University, Xi’an, China

**Keywords:** gastric cancer, pT2N0M0, adjuvant chemotherapy, surgery alone, prognosis

## Abstract

**Background:** There is no global consensus on adjuvant chemotherapy (ACT) for pT2N0M0 gastric cancer. We conducted a retrospective study to reveal the role of ACT in such patients.

**Methods:** Patients with pT2N0M0 gastric cancer who underwent radical resection with D2 lymphadenectomy for primary gastric cancer between January 2012 and May 2016 were included. Kaplan–Meier and Cox regression were used to evaluate overall survival (OS), disease-specific survival (DSS) and predictors of prognosis. Stratified analysis based on high-risk factors was conducted.

**Results:** Of enrolled 307 patients, 111 patients underwent surgery alone and 196 patients received ACT. Surgery alone (HR = 2.913, 95% CI: 1.494-5.682, *p* = 0.002) and total gastrectomy (HR = 2.445, 95% CI: 1.279-4.675, *p* = 0.007) were independently associated with decreased OS. With the median follow-up of 73.1 months, the 5-year OS rate was 87.9% and 5-year DSS rate was 91.8%. Patients receiving ACT showed a better 5-year OS rate (92.9 *vs*. 79.3%, *p* < 0.001) and DSS rate (96.8 vs. 83.0%, *p* < 0.001) than patients underwent surgery alone. Patients receiving monotherapy (*n* = 130) had a relatively poor prognosis compared to patients receiving dual-drug (*n* = 66) without a significant difference (92.3 *vs*. 93.9%, *p* = 0.637). In patients without high-risk factors based on the Chinese Society of Clinical Oncology (CSCO) Guidelines, ACT also provided survival benefit (96.0 vs 82.9%, *p* = 0.038).

**Conclusions:** ACT was accompanied with higher 5-year OS and DSS rates of patients with pT2N0M0 gastric cancer. Patients with pT2N0M0 gastric cancer, regardless of high-risk factors based on the CSCO guidelines, might be considered candidates for ACT. In regard to the therapy regimen, monotherapy might be the optimal choice, considering the adverse events.

## Introduction

Gastric cancer is the fourth leading cause of death from malignant tumors worldwide and the third main cause of cancer death in China (Cao et al., 2021; Navashenaq et al., 2021; Sun et al., 2021; Zeng and Jin, 2021). Despite the incidence of gastric cancer has reduced, gastric cancer related mortality has not changed (Sukri et al., 2020; Varon et al., 2021). Benefiting from advances in medical technology and the popularity of endoscopy, more and more gastric cancer is diagnosed at a relatively early stage. pT2N0M0 gastric cancer is defined as tumors infiltrating the muscularis propria without regional lymph node metastasis or distant metastasis based on the 8^th^ edition of the AJCC TNM staging system for gastric cancer (Amin et al., 2017; Brierley et al., 2017).

Surgery is the only potential chance of cure for gastric cancer, but a certain percentage of patients relapse after curative surgery, which leads to a poor prognosis. Adjuvant chemotherapy (ACT) or chemoradiotherapy has been demonstrated to be beneficial in numerous clinical trials worldwide (Macdonald et al., 2001; Sasako et al., 2011; Noh et al., 2014; Park et al., 2015). Nevertheless, these trials did not report whether patients with less advanced disease would benefit from adjuvant therapy. There are few studies on patients with pT2N0M0 gastric cancer.

Consensus guidelines provide disparate recommendations. Based on the National Comprehensive Cancer Network (NCCN) Guidelines (version 1.2021, Gastric Cancer), options for pT2N0M0 gastric cancer patients after D2 lymph node dissection include surveillance or ACT. Patients with poorly differentiated or high-grade cancer, lymphovascular invasion, neural invasion or aged <50 years are candidates for ACT (National and Comprehensive, 2020). Meanwhile, observation without adjuvant therapy after curative resection is recommended for stage I (including T2N0M0) gastric cancer according to the Japanese Gastric Cancer Treatment Guidelines 2018 (5^th^ edition) (Japanese Gastric Cancer A, 2020). ACT may decrease the risk of metastasis in high-risk pT2N0M0 patients, such as those aged <40 years or with high-grade or poorly differentiated tumor and nervous, lymphovascular invasion, based on the Chinese Society of Clinical Oncology (CSCO) Guidelines (version 1.2021, Gastric Cancer); however, it is unclear whether there is survival benefit of ACT for stage I gastric cancer ((Wang et al., 2021)).

Based on the 8th edition of the TNM staging system of gastric cancer, pT2N0M0 gastric cancer belongs to stage IB, which has good prognosis, with 5-year survival rate of approximately 80–90% after curative surgery ((He et al., 2018; Ji et al., 2018)). The recurrence rates for pT2N0M0 gastric cancer after resection range from 3 to 9% (Jin et al., 2015; Park et al., 2016). Considering the number of patients with stage I gastric cancer is increasing, several retrospective studies focused on the role of ACT in patients with pT2N0M0 gastric cancer and evaluated the high-risk factors of relapse and death; however, they reported diverse opinions on the effect of ACT on pT2N0M0 gastric cancer.

Since it was an open question whether ACT would benefit patients with pT2N0M0 gastric cancer, we aimed to determine the effect of ACT after curative resection in this study.

## Materials and Methods

### Patients

**FIGURE 1:**
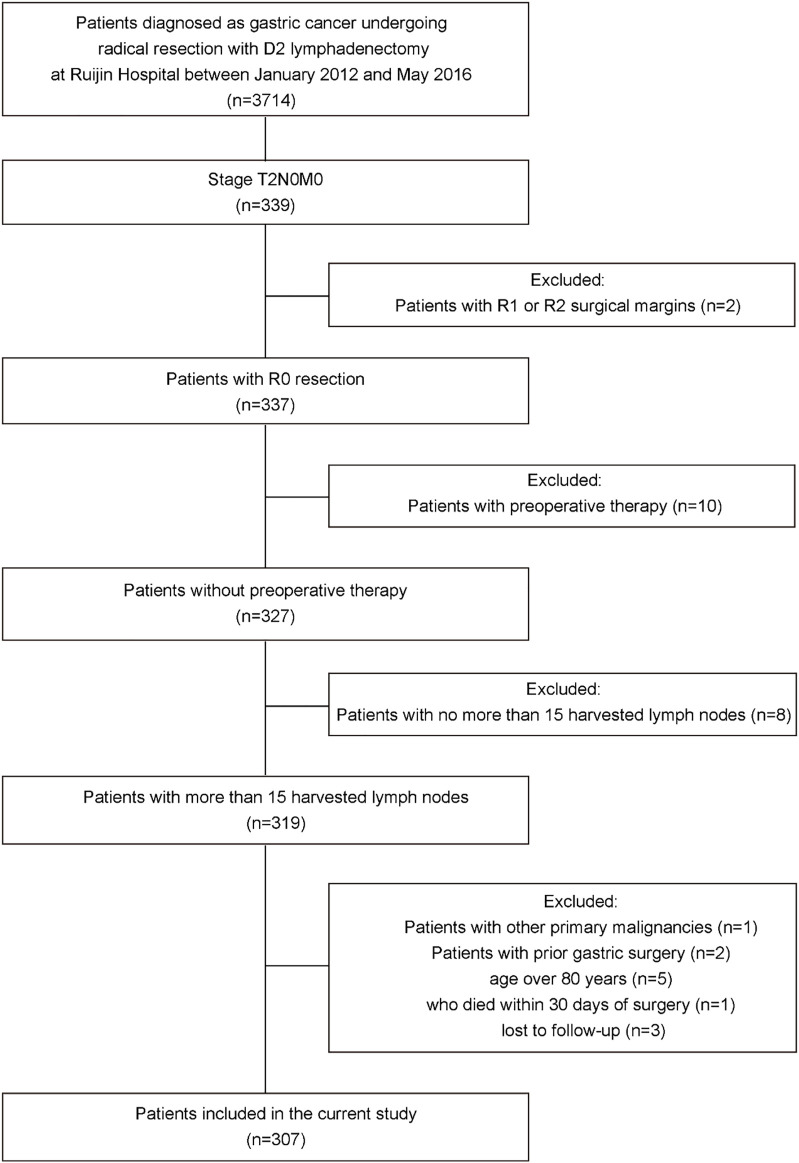
Flowchart of patient selection process.

All patients who underwent radical resection with D2 lymphadenectomy for primary gastric cancer and were ultimately diagnosed with pT2N0M0 gastric cancer based on the 8th edition of the AJCC TNM staging system for gastric cancer at Ruijin Hospital, Shanghai Jiao Tong University School of Medicine between January 2012 and May 2016 were reviewed. Patients with less than 16 harvested lymph nodes, other primary malignancies, prior gastric surgery, R1 or R2 surgical margins, age over 80 years, with postoperative complications; who died within 30 days of surgery; who were lost to follow-up and who received preoperative treatment were excluded. All surgeons had experience doing gastric surgery (>100 procedures per year) and the standard operating procedures were based on the principles of surgery of CSCO Guidelines. Finally, a total of 307 patients were included in this study (Figure 1). This study was approved by the Ruijin Hospital Ethics Committee, Shanghai Jiao Tong University School of Medicine, China (No. 2018-151).

### Evaluation of Clinical Pathological Variables

Clinical pathological characteristics, including age, sex, tumor size, location, Borrmann type, differentiation, histopathology, invasion depth, number of examined lymph nodes, lymphovascular invasion, perineural invasion, resection patterns and treatment regimen after surgery were analyzed. Age was converted to categorical variable, and the cutoff value (40 years) was decided based on the high-risk factors according to the CSCO guidelines (Wang et al., 2019a). Tumor location was classed as the upper, middle, or lower third of the stomach. Tumor histopathology was reviewed based on the WHO classification of the digestive system tumors, 5^th^ edition (WHO Classification of Tumours Editorial Board 2019). Histological type was divided into two groups: differentiated type (including well differentiated and moderately differentiated tubular adenocarcinoma) and undifferentiated type (including mucinous adenocarcinoma, signet ring cell carcinoma and poorly differentiated adenocarcinoma). The tumor invasion depth was divided into the superficial muscularis propria (sMP) layer and the deep muscularis propria (dMP) layer according to pathological examination (Sun et al., 2009). This category was based on the type of muscularis propria fibers; the transverse and longitudinal muscle layers were classified as the sMP and dMP layers, respectively. Lymphovascular invasion was defined as malignant cells appearing in a vascular wall structure or tubular space lined by endothelial cells. Perineural invasion was diagnosed when tumor cells were present in the perineural space of nerves. Total or subtotal gastrectomy was conducted based on the tumor location. Two independent, experienced pathologists reviewed hematoxylin-eosin (H&E)-stained slides from each case. If the diagnosis of the two pathologists was inconsistent, a third pathologist was needed.

### Treatment After Surgery

All patients received postoperative examinations within 3–4 weeks after surgery and patients who received ACT start therapy within 4–6 weeks after surgery. All patients included in my study was with adequate organ function for chemotherapy and PS 0-1. Decisions to administer ACT to patients with pT2N0M0 gastric cancer were based on the preference of surgeons or oncologists. Some doctors approve the Japanese guidelines, thereby they do not recommend postoperative chemotherapy for pT2N0M0 gastric cancer patients; some doctors follow the Chinese guidelines, so they recommend postoperative chemotherapy for patients with high-risk factors based on the CSCO guidelines. Patients with younger age, undifferentiated tumor, perineural or lymphovascular invasion were more likely to receive dual-drug regimen. Patients were given S-1 as monotherapy, while the dual-drug regimen included XELOX or SOX. S-1 was given as follows: 40mg/m2 p. o. b. i.d. day 1-day 14, Q3W for 1 year (Sasako et al., 2011). XELOX was given as six 3-week cycles of capecitabine (1,000 mg/m2 p. o. b. i.d. days 1–14) plus oxaliplatin (130 mg/m2 iv. day 1) (Noh et al., 2014). SOX was given as six 3-week cycles of S-1 (40 mg/m2 p. o. b. i.d. days 1–14) plus oxaliplatin (130 mg/m2 iv. day 1) (Park et al., 2021). Adverse events were assessed by the Common Terminology Criteria for Adverse Events (version 5.0). Dose reduction or interruption were allowed if patients had adverse events of grade 3 or 4. Patients underwent surgery alone accepted no anticancer therapy until recurrence. When cancer relapse was observed, first-line treatment was administered.

### Follow-Up

Outpatient follow-up was conducted every 3 months in the first 2 years and every 6 months for the next 3 years and included a physical examination, blood tests, and tumor markers. Chest-abdomen-pelvis CT and endoscopy were performed every 6 months. Liver MRI, bone scans and PET were optional. The follow-up lasted at least 5 years after surgery or until censoring date or death.

### Statistical Analysis

Continuous variable is shown as median with interquartile ranges (IQRs), and categorical variable is presented as number with proportions. Categorical variable was analyzed using Fisher’s exact test or chi square test. DSS was defined as the time of surgery to death from gastric cancer. The 5-year OS and DSS rates were calculated using the Kaplan-Meier curve, and differences were analyzed by the log-rank test. Independent predictors of survival were found by Cox-regression survival analysis. Hazard ratio (HR) > 1 was related to a higher hazard of death. A *p* value < 0.05 was considered as statistically significant. SPSS version 22.0 for Windows (IBM Corporation, Armonk, NY, United States) was used for statistical analysis.

## Results

### Clinical Pathological Features of Patients With pT2N0M0 Gastric Cancer

A total of 307 patients with pT2N0M0 gastric cancer were enrolled in this study. The clinical pathological characteristics are shown in Table 1. The age ranged from 29 to 80 years, with a median age of 63 years. Most of patients were male (*n* = 216, 70.4%). The median tumor size was 2.5 cm. Size was converted to categorical variable, and the cutoff value was median size. Tumors were more likely located in the lower 1/3 of the stomach (*n* = 206, 67.1%) and presented as the Borrmann III type (*n* = 151, 51.1%) and undifferentiated type (n = 211, 68.7%). The median number of harvested lymph nodes was 22 with a range from 16 to 68. Twenty-three patients had perineural invasion and 33 patients had lymphovascular invasion. 63.8% of patients received ACT, 130 patients received monotherapy and 66 patients were given dual drug treatment.

**TABLE 1:** Clinical pathological characteristics of patients with pT2N0M0 gastric cancer.

Variables	Total (*n* = 307)	With high-risk factors (*n* = 216)	Without high-risk factors (*n* = 91)	*p* Value
Age (years)				0.022*
Median (IQRs)	63 (56, 71)	63 (55, 71)	64 (60, 71)	
<40	12 (3.9)	12 (5.6)	0	
≥40	295 (96.1)	204 (94.4)	91 (100)	
Sex				0.006*
Male	216 (70.4)	142 (65.7)	74 (81.3)	
Female	91 (29.6)	74 (34.3)	17 (18.7)	
Location				0.913
Upper	55 (17.9)	40 (18.5)	15 (16.5)	
Middle	46 (15.0)	32 (14.8)	14 (15.4)	
Lower	206 (67.1)	144 (66.7)	62 (68.1)	
Size (cm)				0.989
≤2.5	155 (50.5)	109 (50.5)	46 (50.5)	
>2.5	152 (49.5)	107 (49.5)	45 (49.5)	
Borrmann				0.661
I	39 (12.7)	26 (12.0)	13 (14.3)	
II	111 (36.2)	76 (35.2)	35 (38.5)	
III	157 (51.1)	114 (52.8)	43 (47.3)	
Differentiation				<0.001*
Differentiated	96 (31.3)	5 (2.3)	91 (100)	
Undifferentiated	211 (68.7)	211 (97.7)	0	
Histopathology				<0.001*
Tub	96 (31.3)	5 (2.3)	91 (100)	
Por	160 (52.1)	160 (74.1)	0	
Sig	32 (10.4)	32 (14.8)	0	
Muc	19 (6.2)	19 (8.8)	0	
Depth				0.356
sMP	163 (53.1)	111 (51.4)	52 (57.1)	
dMP	144 (46.9)	105 (48.6)	39 (42.9)	
Examined LNs (Median (IQRs))	22 (18, 29)	21 (18, 29)	23 (18, 28)	0.425
PNI	23 (7.5)	23 (10.6)	0	0.001*
LVI	33 (10.7)	33 (15.3)	0	<0.001*
Gastrectomy				0.652
Distal	224 (73.0)	156 (72.2)	68 (74.7)	
Total	83 (27.0)	60 (27.8)	23 (25.3)	
Postoperative treatment				0.035*
ACT	196 (63.8)	146 (67.6)	50 (54.9)	
SA	111 (36.2)	70 (32.4)	41 (45.1)	
ACT type				0.018*
Monotherapy	130 (66.3)	90 (61.6)	40 (80.0)	
Dual drug	66 (33.7)	56 (38.4)	10 (20.0)	

*p* < 0.05 was considered statistically significant.

High-risk factors including patients aged <40 years or with high-grade or poorly differentiated tumor and nervous, lymphovascular invasion, according to the CSCO Guidelines (version 1.2018, Gastric Cancer); Tub, tubular adenocarcinoma; Por, poorly differentiated adenocarcinoma; Sig, signet ring cell carcinoma; Muc, mucinous adenocarcinoma; sMP, superficial muscularis propria layer; dMP, deep muscularis propria layer; LNs, lymph nodes; IQRs, interquartile ranges; PNI, perineural invasion; LVI, lymphovascular invasion; ACT, adjuvant chemotherapy; SA, surgery alone.

### Long-Term Outcomes and Effect of ACT on Prognosis in pT2N0M0 Gastric Cancer Patients

**FIGURE 2:**
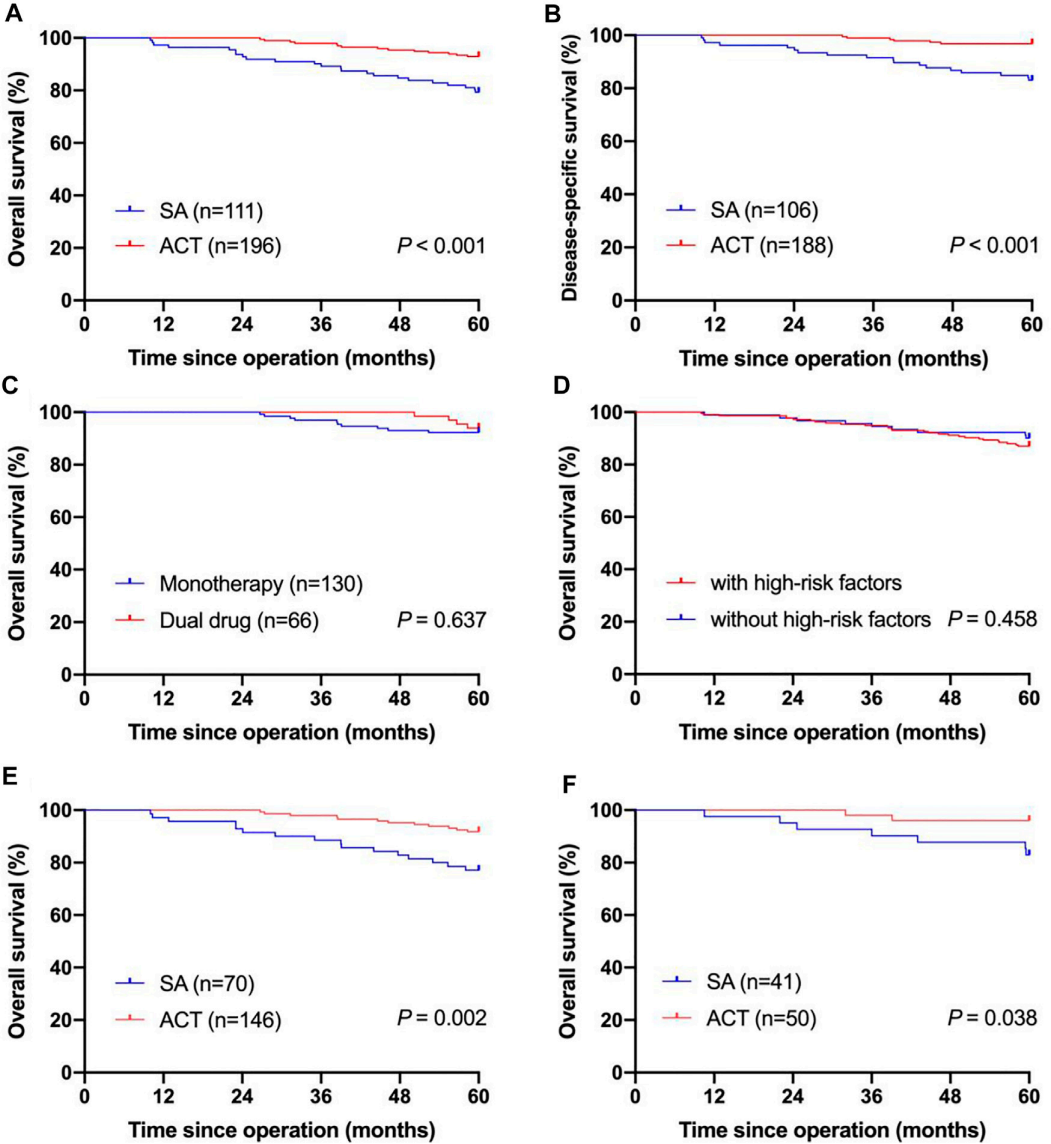
Kaplan-Meier curves for survival of pT2N0M0 gastric cancer patients. **(A)** Kaplan-Meier curves of pT2N0M0 gastric cancer patients underwent SA and patients receiving ACT in the OS analysis; **(B)** Kaplan-Meier curves of pT2N0M0 gastric cancer patients underwent SA and patients receiving ACT in the DSS analysis; **(C)** Kaplan-Meier curves of pT2N0M0 gastric cancer patients who received monotherapy and patients who received the dual-drug regimen in the OS analysis; **(D)** Kaplan-Meier curves of pT2N0M0 gastric cancer patients with high-risk factors and patients without high-risk factors in the OS analysis; **(E)** Kaplan-Meier curves of pT2N0M0 gastric cancer patients with high-risk factors who underwent SA and who received ACT in the OS analysis; **(F)** Kaplan-Meier curves of pT2N0M0 gastric cancer patients without high-risk factors who underwent SA and who received ACT in the OS analysis; SA, surgery alone; ACT, adjuvant chemotherapy; OS, overall survival; DSS, disease-specific survival.

As of May 2021, the median follow-up was 73.1 months, ranging from 10 to 112.9 months. In our study, 5-year OS rate of all patients was 87.9%. Kaplan–Meier survival analysis showed that the 5-year OS rate was higher in patients who received ACT (92.9%) compared with those who underwent surgery alone (79.3%, *p* < 0.001, Figure 2A). In the Cox-regression analysis, independent predictors of decreased OS were surgery alone (HR = 2.913, 95% CI: 1.494-5.682, *p* = 0.002) and total gastrectomy (HR = 2.445, 95% CI: 1.279-4.675, *p* = 0.007, Table 2).

**TABLE 2:** Univariate and Cox-regression of overall survival of patients with pT2N0M0 gastric cancer.

Variables	5-year OS rate (%)	*p* Value	Cox-regression		
			HR	95% CI	*p* Value
Age (years)		0.665			
<40	91.7
≥40	87.8
Sex		0.126
Male	86.1
Female	92.3
Location		0.773
Upper	85.5
Middle	87.0
Lower	88.8
Size (cm)		0.399
≤2.5	86.5
>2.5	89.5
Borrmann		0.627
I	89.7
II	85.6
III	89.2
Differentiation		0.339
Differentiated	90.6
Undifferentiated	86.7
Histopathology		0.776
Tub	90.6
Por	86.3
Sig	87.5
Muc	89.5
Depth		0.327
sMP	89.6
dMP	86.1
Examined LNs		0.556
LVI		0.273
Negative	87.2
Positive	93.9
PNI		0.166
Negative	88.7
Positive	78.3
Gastrectomy		0.001*
Distal	91.5
Total	78.3		2.445	1.279-4.675	0.007*
Postoperative treatment		<0.001*
SA	79.3		2.913	1.494-5.682	0.002*
ACT	92.9				
ACT type		0.637			
Monotherapy	92.3				
Dual drug	93.9				

*p < 0.05 was considered statistically significant.

Tub, tubular adenocarcinoma; Por, poorly differentiated adenocarcinoma; Sig, signet ring cell carcinoma; Muc, mucinous adenocarcinoma; sMP, superficial muscularis propria layer; dMP, deep muscularis propria layer; LNs, lymph nodes; PNI, perineural invasion; LVI, lymphovascular invasion; ACT, adjuvant chemotherapy; SA, surgery alone; HR, hazard ratio; 95% CI, 95% confidence interval.

The 5-year DSS rate of enrolled patients was 91.8% when excluding 13 patients who did not die from gastric cancer. Supplementary Table S1 shows the clinical pathological characteristics of patients enrolled in the DSS analysis. Patients receiving ACT showed a better 5-year DSS rate (96.8 vs. 83.0%, *p* < 0.001) than patients underwent surgery alone with significant difference (Figure 2B). In the Cox-regression analysis, surgery alone (HR = 5.052, 95% CI: 1.993-12.809, *p* = 0.001) and total gastrectomy (HR = 2.820, 95% CI: 1.256-6.329, *p* = 0.012) were independently associated with decreased OS (Supplementary Table S2). Supplementary Table S4 shows the dominant recurrence sites in patients who died of gastric cancer relapse.

### Effect of the ACT Regimen on Prognosis in pT2N0M0 Gastric Cancer

Of 196 patients received ACT, 130 patients received monotherapy, and 66 patients received dual-drug chemotherapy. Supplementary Table S3 shows the clinical pathological variables of patients who were given different chemotherapy regimens. The clinical pathological characteristics between the two groups were comparable, except for age, differentiation, lymphovascular invasion and perineural invasion. Most patients with lymphovascular invasion or perineural invasion received dual-drug chemotherapy, and the 5-year OS rate of the dual-drug subgroup reached 93.9%, while the monotherapy subgroup had a relatively poor prognosis, without a significant difference (92.3%, *p* = 0.637, Figure 2C).

Grade 5 adverse events did not occur. The main grade 3 or 4 adverse events were anemia (9.2%), anorexia (6.9%) diarrhea (4.6%) in the monotherapy group and neutropenia (15.2%), peripheral neuropathy (12.1%), anorexia (7.6%) and anemia (4.5%) in the dual-drug regimen group.

### Stratification by High-Risk Factors According to the CSCO Guidelines

According to the CSCO guidelines, high-risk factors include patients aged <40 years or with high-grade or poorly differentiated tumor and nervous, lymphovascular invasion. Seventy-three patients had high-risk factors and thirty-four patients did not have high-risk factors. The clinical pathological features of patients stratified by high-risk factors was showed in Table 1. The 5-year OS rate was lower in patients with high-risk factors (87.0%) compared with those without high-risk factors (90.1%), whereas the difference was not statistically significant (*p* = 0.458, Figure 2D).

In patients with high-risk factors, gastrectomy type and postoperative therapy were concerned with prognosis in the univariate analysis. In patients with high-risk factors, the 5-year OS rate of patients received ACT was significantly higher than that of patients underwent surgery alone (91.8 vs 77.1%, *p* = 0.002, Figure 2E). In the Cox-regression analysis, surgery alone (HR = 3.130, 95% CI: 1.480-6.620, *p* = 0.003) and total gastrectomy (HR = 3.303, 95% CI: 1.571-6.947, *p* = 0.002) were independently associated with decreased OS (Table 3). In patients without high-risk factors, the 5-year OS rate of patients received ACT was also significantly higher than that of patients underwent surgery alone (96.0 vs 82.9%, *p* = 0.038, Figure 2F). Thus, ACT could not only increase the 5-year survival rate of patients with high-risk factors, but also increase the 5-year survival rate of patients without high-risk factors.

**TABLE 3:** Univariate and Cox-regression of overall survival of patients with pT2N0M0 gastric cancer stratified by high-risk factors.

Variables	With high-risk factors					Without high-risk factors	
	5-year OS rate	*P* value	Cox-regression			5-year OS rate	*P* value
			HR	95% CI	*P* value		
Age (years)		0.601				—	—
< 40	91.7%						
≥ 40	86.8%						
Sex		0.122					0.534
Male	84.5%					89.2%	
Female	91.9%					94.1%	
Location		0.479					0.797
Upper	82.5%					93.3%	
Middle	84.4%					92.9%	
Lower	88.9%					88.7%	
Size (cm)		0.420					0.754
≤ 2.5	85.3%					89.1%	
> 2.5	88.8%					91.1%	
Borrmann		0.654					0.311
I	92.3%					84.6%	
II	85.5%					85.7%	
III	86.8%					95.3%	
Differentiation		0.399				—	—
Differentiated	100%						
Undifferentiated	86.7%						
Histopathology		0.829				—	—
Tub	100%						
Por	86.3%						
Sig	87.5%						
Muc	89.5%						
Depth		0.536					0.410
sMP	88.3%					92.3%	
dMP	85.7%					87.2%	
Examined LNs		0.927					0.210
LVI		0.211				—	—
Negative	85.8%						
Positive	93.9%						
PNI		0.223				—	—
Negative	88.1%						
Positive	78.3%						
Gastrectomy		0.001*					0.552
Distal	91.7%					91.2%	
Total	75.0%		3.303	1.571-6.947	0.002*	87.0%	
Postoperative treatment		0.002*					0.038*
SA	77.1%		3.130	1.480-6.620	0.003*	82.9%	
ACT	91.8%					96.0%	
ACT type		0.664					0.477
Monotherapy	91.1%					95.0%	
Dual drug	92.9%					100%	

*p < 0.05 was considered statistically significant.

High-risk factors including patients aged <40 years or with high-grade or poorly differentiated tumor and nervous, lymphovascular invasion, according to the CSCO Guidelines (version 1.2018, Gastric Cancer); Tub, tubular adenocarcinoma; Por, poorly differentiated adenocarcinoma; Sig, signet ring cell carcinoma; Muc, mucinous adenocarcinoma; sMP, superficial muscularis propria layer; dMP, deep muscularis propria layer; LNs, lymph nodes; PNI, perineural invasion; LVI, lymphovascular invasion; ACT, adjuvant chemotherapy; SA, surgery alone; HR, hazard ratio; 95% CI, 95% confidence interval.

In patients without high-risk factors, 40 patients received monotherapy, 10 patients received dual-drug regimen. The 5-year OS rate was 95.0% for the monotherapy subgroup and 100% for the dual-drug subgroup without significant difference (*p* = 0.477, Table 3). In patients with high risk factors, 90 patients received monotherapy, 56 patients received dual-drug regimen. The 5-year OS rate was 91.1% for the monotherapy subgroup and 92.9% for the dual-drug subgroup without significant difference (*p* = 0.664, Table 3).

## Discussion

ACTS-GC trial (Sasako et al., 2011) demonstrated that patients with stage II/III gastric cancer could significantly benefit from adjuvant S-1. CLASSIC trial (Noh et al., 2014) also showed survival benefit of adjuvant XELOX for stage II/III gastric cancer patients. ARTIST II trial (Park et al., 2021) showed that adjuvant SOX was more effective than S-1 in patients with node positive, stage II/III gastric cancer. Exiting prospective randomized clinical trials demonstrating the benefit of ACT could not explain whether all gastric cancer patients (especially stage IB gastric cancer) would benefit from ACT. Although the prognosis of pT2N0M0 gastric cancer is relatively good in general, postoperative relapse still occurs in some patients with various recurrence sites.

In the current study, we found a good prognosis of pT2N0M0 gastric cancer, with the 5-year OS rate of 87.9% and 5-year DSS rate of 91.8%, similar to other studies ((In et al., 2016; Park et al., 2016)).

Some retrospective studies identified risk factors in stage I gastric cancer patients. The authors of a Korean study focusing on stage I gastric cancer reported that age, sex, stage IB, lymphatic vessel invasion, nerve invasion and a high serum carcinoembryonic antigen level, were independent prognostic factors (Caccialanza et al., 2016; Liu et al., 2016). A population-based study using the Surveillance, Epidemiology, and End Results (SEER) database demonstrated that older age, proximal tumor location, high tumor grade and large tumor size were independent factors of poor disease-related survival (Gold et al., 2013). Other studies found that several clinical pathological factors were significantly associated with a high risk of relapse and death in pT2N0M0 gastric cancer patients and suggested that patients with high-risk factors receive ACT. A further Chinese study identified the upper 1/3 of the stomach, large tumor diameter, perineural and lymphovascular invasion as independent risk factors associated with decreased OS rates (Wang et al., 2018). Another study also reported that lymphatic vessel and nerve invasion and tumor size were independent risk factors (Caccialanza et al., 2016; Liu et al., 2016).

Our study found that total gastrectomy and surgery alone were independent risk factors for survival. Other studies also found many other risk factors associated with a poor prognosis. The main reason for this inconsistency was study heterogeneity, with differences in race, surgical practice and initial prognosis.

A single-center study from the CLASSIC trial (Caccialanza et al., 2016; Liu et al., 2016) found a marked loss in body composition parameters (muscle, visceral fat and subcutaneous fat) significantly predicted short disease-free survival and OS among patients who underwent gastrectomy. Malnutrition was considered as poor prognostic factor in cancer patients (Caccialanza et al., 2016; Liu et al., 2016). Fujiya demonstrated that persistent postoperative malnutrition was frequently observed in patients who underwent total gastrectomy (Fujiya et al., 2018). These studies might explain why patients who received total gastrectomy had poor prognoses in our study, although we could not evaluate the nutrition index.

Despite a lack of prospective studies that explored the benefit of ACT in less advanced gastric cancer, there were some retrospective studies exploring the effect of ACT such patients. Based on the 8th edition of the TNM staging system of gastric cancer, stage IB gastric cancer includes pT1N1M0 and pT2N0M0. Wang used the SEER database to explore the difference between T1N1M0 and T2N0M0 and found that patients with T2N0M0 gastric cancer may not benefit from adjuvant treatment (Wang et al., 2019b). Recently, Jin et al.(Jin et al., 2021) found that pT2N0 gastric cancer patients with non-signet ring cell carcinoma, tumor size >3 cm and examined lymph nodes≤15 may be particularly appropriate candidates for ACT. In our study, there was no significant difference in OS between patients with signet ring cell carcinoma and patients with other histopathology type.

Since 1997, the retrieval of at least 15 lymph nodes has been recommended for adequate gastric cancer staging, and several studies have found that lymphadenectomy with <15 lymph nodes removed was an adverse independent prognostic factor for OS. A SEER study demonstrated that OS was dependent on the number of harvested lymph nodes; in patients with node-negative T1-2 gastric cancer, every additional 10 lymph nodes harvested increased the 5-year survival rate of 7.6% (Smith et al., 2005). Haejin found that their subgroup of T2N0M0 gastric cancer patients who underwent suboptimal lymphadenectomy benefitted from chemoradiotherapy rather than chemotherapy (Coburn et al., 2008; Du et al., 2011). Due to a lack of patients who received postoperative radiotherapy, the differences in radiotherapy and chemoradiotherapy roles could not be established in our study. Other studies failed to show the number of removed lymph nodes as an independent prognostic factor (Coburn et al., 2008; Du et al., 2011). One large population-based study demonstrated that surgery with adequate lymph node removing alone (≥15 lymph nodes) predicted better prognosis compared with adjuvant therapy in patients with stage I or node-negative gastric cancer (Dudeja et al., 2012). Our study found that the number of harvested lymph nodes was not associated with prognosis, which may be related to excluding patients with fewer than 15 harvested lymph nodes.

Several studies on patients with pT2 gastric cancer focused on the invasion depth. Some studies have showed that pT2 gastric cancer patients showing invasion into dMP had a relatively poor prognosis than those only invasion sMP (Zhang et al., 2016; Park et al., 2021), while others reported no significant difference in the prognosis between the two groups (Son et al., 2007; Nakamura et al., 2019). In our study, the difference in the 5-year OS rate between the sMP and dMP subgroups was not significant (89.6 *vs*. 86.1%, *p* = 0.327).

Regarding the therapy regimen, monotherapy and dual-drug therapy showed no significant difference. ACTS-GC trial and CLASSIC trial demonstrated that ACT with S-1 or XELOX was safe. In our study, the main grade 3 or 4 adverse events were neutropenia, peripheral neuropathy in the dual-drug group and anemia, anorexia in the monotherapy group. According to the ACTS-GC trial ((Sakuramoto et al., 2007)), the most common adverse events of grade 3 or grade 4 were anorexia (6.0%), nausea (3.7%), and diarrhea (3.1%) in the S-1 group. According to the CLASSIC trial ((Bang et al., 2012)), the main grade 3 or 4 adverse events were neutropenia (22%), thrombocytopenia (8%), nausea (8%), and vomiting (7%) in the XELOX group. According the ARTIST II study ((Nagaraja et al., 2019; Li et al., 2020; Low et al., 2021; Nakazawa et al., 2021)), the most common adverse events of grade 3 or 4 were peripheral neuropathy (12%), anemia (8%) and anorexia (4%) in the SOX group. The common dose-limiting toxicity of oxaliplatin is peripheral neuropathy, which affects 90% patients ((Kweekel et al., 2005)). The incidence of peripheral neuropathy is considered to be related to the prolonged use of oxaliplatin ((Baek et al., 2010)). Thus, we recommend monotherapy to prevent toxicity and discomfort. However, other studies, which aim to explore the role of ACT in stage IB gastric cancer, failed to analyse the difference between monotherapy and dual-drug therapy.

According to the CSCO guidelines, patients with pT2N0M0 gastric cancer with high-risk factors (age <40 years or with high-grade or poorly differentiated tumor and nervous, lymphovascular invasion) are recommended to receive ACT to reduce the risk of recurrence. Then, we divided patients with pT2N0M0 gastric cancer into two subgroups (with high-risk factors and without high-risk factors) and evaluated whether the effect of postoperative therapy was diverse. ACT indeed provided survival benefits to patients with high-risk factors, while patients without high-risk factors also benefitted from ACT, which was inconsistent with the CSCO guidelines. Regarding the therapy regimen, monotherapy and dual-drug therapy showed no significant difference; thus, considering possible adverse events, we recommend monotherapy regardless of high-risk factors. There were no studies exploring the role of ACT stratified by high-risk factors based on the CSCO guidelines.

Although patients with gastric cancer received ACT after radical gastrectomy, some patients still experienced relapse. Timely detection of recurrence, as well as identification of patients at high risk of relapse after surgery or completion of adjuvant therapy are major challenges in the treatment of gastric cancer. Drug resistance is the major factor of treatment failure and relapse and numerous studies aim to investigate the mechanisms of drug resistance ((Nagaraja et al., 2019; Li et al., 2020; Low et al., 2021; Nakazawa et al., 2021)). Over the past few decades, predictive biomarkers have received increasing attention in diagnosis, treatment, and prognosis of gastric cancer. Studies have found many predictive biomarkers for the precision treatment of gastric cancer (Petrillo and Smyth, 2020). In 2014, The Cancer Genome Atlas (Cancer Genome Atlas Resea, 2014) proposed a molecular classification of gastric cancer into 4 subtypes: chromosomal instability, Epstein-Barr virus positive, genomically stable and microsatellite instability (MSI). An et al.(An et al., 2012) found that in stage II/III gastric cancer, patients with microsatellite stable and MSI-low type significantly benefited from 5-FU-based ACT, while patients with MSI-high type did not benefit from 5-FU-based ACT. Findings from the MAGIC trial (Smyth et al., 2017) showed that mismatch repair deficiency (dMMR) and MSI-high were associated with good prognosis in patients treated with surgery alone, whereas in gastric cancer patients treated with perioperative chemotherapy, dMMR and MSI-high were associated with worse prognosis. Post hoc analysis of CLASSIC trial (Choi et al., 2019) showed that MSI-high was independent prognostic factor and ACT significantly improved disease-free survival in MSS group while no benefit was found in the MSI-high group. MSI status could be used for precision treatment of gastric cancer in the future.

A prospective randomized trial comparing surgery alone with ACT in stage IB gastric cancer patients with at least one risk factor for recurrence (male sex, age>65 years, perineural and lymphovascular invasion) is now ongoing (ClinicalTrials.gov identifier NCT01917552), and this large-scale prospective trial is expected to compensate for previous research shortcomings and yield satisfactory results. Although the trial is based on the 6th edition of the AJCC staging system, it also includes pT2N0M0 gastric cancer based on the 8th edition of the AJCC staging system.

Nevertheless, there are several potential limitations in this study. The number of patients with pT2N0M0 gastric cancer was relatively small since it was a single-center study, the resultant effects may have been underestimated, and the results should be interpreted with caution. In addition, this was a retrospective study, and there were likely patient and tumor baseline characteristic imbalances between the treatment groups. Finally, the role of radiotherapy was not analysed due to a lack of patients who received postoperative radiotherapy. Therefore, the conclusions of this study need to be verified by prospective study with a large sample size.

## Conclusion

ACT was accompanied with higher 5-year OS and DSS rates of patients with pT2N0M0 gastric cancer. Patients with pT2N0M0 gastric cancer, regardless of high-risk factors based on the CSCO guidelines, might be considered candidates for ACT. In regard to the therapy regimen, monotherapy might be the optimal choice, considering the adverse events.

## Data Availability

The original contributions presented in the study are included in the article/Supplementary Material, further inquiries can be directed to the corresponding authors.
