# Supplementary material for: Adjuvant Chemotherapy in pT2N0M0 Gastric Cancer: Findings From a Retrospective Study
Source: Front Pharmacol. 2022 Feb 17;13:845261. doi: 10.3389/fphar.2022.845261 (PMC8891981; doi:10.3389/fphar.2022.845261)
Supplement: Supplementary file 1 [file DataSheet1.docx]

**Table S1. Clinical pathological characteristics of pT2N0M0 gastric cancer patients enrolled in the DSS analysis.**

| Variables | Total  (n=294) | SA  (n=106) | ACT  (n=188) | *P* value |
| --- | --- | --- | --- | --- |
| Age (years) |  |  |  | 0.983 |
| <40 | 11 (3.7) | 4 (3.8) | 7 (3.7) |  |
| ≥40 | 283 (96.3) | 102 (96.2) | 181 (96.3) |  |
| Sex |  |  |  | 0.153 |
| Male | 207 (70.4) | 80 (75.5) | 127 (67.6) |  |
| Female | 87 (29.6) | 26 (25.2) | 61 (32.4) |  |
| Location |  |  |  | 0.480 |
| Upper | 53 (18.0) | 22 (20.8) | 31 (16.5) |  |
| Middle | 45 (15.3) | 18 (17.0) | 27 (14.4) |  |
| Lower | 196 (66.7) | 66 (62.3) | 130 (69.1) |  |
| Size (cm) |  |  |  | 0.627 |
| ≤2.5 | 147 (50.0) | 51 (48.1) | 96 (51.1) |  |
| >2.5 | 147 (50.0) | 55 (51.9) | 92 (48.9) |  |
| Borrmann |  |  |  | 0.607 |
| I | 38 (12.9) | 16 (15.1) | 22 (11.7) |  |
| II | 105 (35.7) | 39 (36.8) | 66 (35.1) |  |
| III | 151 (51.4) | 51 (48.1) | 100 (53.2) |  |
| Differentiation |  |  |  | 0.112 |
| Differentiated | 94 (32.0) | 40 (37.7) | 54 (28.7) |  |
| Undifferentiated | 200 (68.0) | 66 (62.3) | 134 (71.3) |  |
| Histopathology |  |  |  | 0.321 |
| Tub | 94 (32.0) | 40 (37.7) | 54 (28.7) |  |
| Por | 152 (51.7) | 53 (50.0) | 99 (52.7) |  |
| Sig | 30 (10.2) | 8 (7.5) | 22 (11.7) |  |
| Muc | 18 (6.1) | 5 (4.7) | 13 (6.9) |  |
| Depth |  |  |  | 0.854 |
| sMP | 156 (53.1) | 57 (53.8) | 99 (52.7) |  |
| dMP | 138 (46.9) | 49 (46.2) | 89 (47.3) |  |
| PNI | 20 (6.8) | 6 (5.7) | 14 (7.4) | 0.559 |
| LVI | 32 (10.9) | 7 (6.6) | 25 (13.3) | 0.077 |
| Gastrectomy |  |  |  | 0.106 |
| Distal | 216 (73.5) | 72 (67.9) | 144 (76.6) |  |
| Total | 78 (26.5) | 34 (32.1) | 44 (23.4) |  |

Tub, tubular adenocarcinoma; Por, poorly differentiated adenocarcinoma; Sig, signet ring cell carcinoma; Muc, mucinous adenocarcinoma; sMP, superficial muscularis propria layer; dMP, deep muscularis propria layer; PNI, perineural invasion; LVI, lymphovascular invasion; SA, surgery alone; ACT, adjuvant chemotherapy.

**Table S2. Univariate and Cox-regression of DSS of pT2N0M0 gastric cancer patients**

| Variables | 5-year DSS rate | *P* value | Cox-regression | | |
| --- | --- | --- | --- | --- | --- |
|  |  |  | HR | 95% CI | *P* value |
| Age (years) |  | 0.324 |  |  |  |
| <40 | 100% |  |  |  |  |
| ≥40 | 91.5% |  |  |  |  |
| Sex |  | 0.059 |  |  |  |
| Male | 89.9% |  |  |  |  |
| Female | 96.6% |  |  |  |  |
| Location |  | 0.414 |  |  |  |
| Upper | 88.7% |  |  |  |  |
| Middle | 88.9% |  |  |  |  |
| Lower | 93.4% |  |  |  |  |
| Size (cm) |  | 0.663 |  |  |  |
| ≤2.5 | 91.2% |  |  |  |  |
| >2.5 | 92.5% |  |  |  |  |
| Borrmann |  | 0.818 |  |  |  |
| I | 92.1% |  |  |  |  |
| II | 90.5% |  |  |  |  |
| III | 92.7% |  |  |  |  |
| Differentiation |  | 0.747 |  |  |  |
| Differentiated | 92.6% |  |  |  |  |
| Undifferentiated | 91.5% |  |  |  |  |
| Histopathology |  | 0.913 |  |  |  |
| Tub | 92.6% |  |  |  |  |
| Por | 90.8% |  |  |  |  |
| Sig | 93.3% |  |  |  |  |
| Muc | 94.4% |  |  |  |  |
| Depth |  | 0.237 |  |  |  |
| sMP | 93.6% |  |  |  |  |
| dMP | 89.9% |  |  |  |  |
| LVI |  | 0.272 |  |  |  |
| Negative | 91.2% |  |  |  |  |
| Positive | 96.9% |  |  |  |  |
| PNI |  | 0.770 |  |  |  |
| Negative | 92.0% |  |  |  |  |
| Positive | 90.0% |  |  |  |  |
| Gastrectomy |  | 0.001* |  |  |  |
| Distal | 94.9% |  |  |  |  |
| Total | 83.3% |  | 2.820 | 1.256-6.329 | 0.012* |
| Postoperative therapy |  | <0.001* |  |  |  |
| SA | 83.0% |  | 5.052 | 1.993-12.809 | 0.001* |
| ACT | 96.8% |  |  |  |  |
| ACT type |  | 0.083 |  |  |  |
| Monotherapy | 95.2% |  |  |  |  |
| Dual drug | 100.0% |  |  |  |  |

*P < 0.05 was considered statistically significant; Tub, tubular adenocarcinoma; Por, poorly differentiated adenocarcinoma; Sig, signet ring cell carcinoma; Muc, mucinous adenocarcinoma; sMP, superficial muscularis propria layer; dMP, deep muscularis propria layer; LVI, lymphovascular invasion; PNI, perineural invasion; SA, surgery alone; ACT, adjuvant chemotherapy; HR, hazard ratio; 95% CI, 95% confidence interval.

**Table S3. Clinical pathological characteristics of patients in the monotherapy and dual-drug regimen groups**

| Variables | Total  (n=196) | Monotherapy  (n=130) | Dual drug  (n=66) | *P* value |
| --- | --- | --- | --- | --- |
| Age (years) |  |  |  | 0.001* |
| <40 | 8 (4.1) | 1 (0.8) | 7 (10.6) |  |
| ≥40 | 188 (95.9) | 129 (99.2) | 59 (89.4) |  |
| Sex |  |  |  | 0.061 |
| Male | 133 (67.9) | 94 (72.3) | 39 (59.1) |  |
| Female | 63 (32.1) | 36 (27.7) | 27 (40.9) |  |
| Location |  |  |  | 0.142 |
| Upper | 33 (16.8) | 23 (17.7) | 10 (15.2) |  |
| Middle | 28 (14.3) | 14 (10.8) | 14 (21.2) |  |
| Lower | 135 (68.9) | 93 (71.5) | 42 (63.6) |  |
| Size (cm) |  |  |  | 0.363 |
| ≤2.5 | 101 (51.5) | 70 (53.8) | 31 (47.0) |  |
| >2.5 | 95 (48.5) | 60 (46.2) | 35 (53.0) |  |
| Borrmann |  |  |  | 0.199 |
| I | 23 (11.7) | 15 (11.5) | 8 (12.1) |  |
| II | 70 (35.7) | 52 (40.0) | 18 (27.3) |  |
| III | 103 (52.6) | 63 (48.5) | 40 (60.6) |  |
| Differentiation |  |  |  | 0.036* |
| Differentiated | 54 (27.6) | 42 (32.3) | 12 (18.2) |  |
| Undifferentiated | 142 (72.4) | 88 (67.7) | 54 (81.8) |  |
| Histopathology |  |  |  | 0.165 |
| Tub | 54 (27.6) | 42 (32.3) | 12 (18.2) |  |
| Por | 104 (53.1) | 64 (49.2) | 40 (60.6) |  |
| Sig | 24 (12.2) | 14 (10.8) | 10 (15.2) |  |
| Muc | 14 (7.1) | 10 (7.7) | 4 (6.1) |  |
| Depth |  |  |  | 0.223 |
| sMP | 104 (53.1) | 73 (56.2) | 31 (47.0) |  |
| dMP | 92 (46.9) | 57 (43.8) | 35 (53.0) |  |
| PNI | 16 (8.2) | 6 (4.6) | 10 (15.2) | 0.011* |
| LVI | 26 (13.3) | 10 (7.7) | 16 (24.2) | 0.001* |
| Gastrectomy |  |  |  | 0.951 |
| Distal | 149 (76.0) | 99 (76.2) | 50 (75.8) |  |
| Total | 47 (24.0) | 31 (23.8) | 16 (24.2) |  |

*P < 0.05 was considered statistically significant; Tub, tubular adenocarcinoma; Por, poorly differentiated adenocarcinoma; Sig, signet ring cell carcinoma; Muc, mucinous adenocarcinoma; sMP, superficial muscularis propria layer; dMP, deep muscularis propria layer; PNI, perineural invasion; LVI, lymphovascular invasion.

**Table S4. Recurrence sites in pT2N0M0 gastric cancer patients who died of gastric cancer relapse.**

| Recurrence site | Patients receiving SA | Patients receiving ACT | Total |
| --- | --- | --- | --- |
| Hematogenous | 6 | 1 | 7 |
| Lymph node | 6 | 3 | 9 |
| Peritoneal | 4 | 1 | 5 |
| Unknown site | 2 | 1 | 3 |
| Total | 18 | 6 | 24 |

SA, surgery alone; ACT, adjuvant chemotherapy
